# Highly Sensitive Dual Parameter Sensor Based on a Hybrid Structure with Multimode Interferometer and Fiber Bragg Grating Fabricated by Femtosecond Laser

**DOI:** 10.3390/s21175938

**Published:** 2021-09-03

**Authors:** Xinran Dong, Li Zeng, Dongkai Chu, Xiaoyan Sun

**Affiliations:** 1 College of Mechanical and Electrical Engineering, Central South University of Forestry and Technology, 498 South Shaoshan Street, Changsha 410004, China; xrdong@csuft.edu.cn; 2 State Key Laboratory of High Performance Complex Manufacturing, College of Mechanical and Electrical Engineering, Central South University, 932 South Lushan Street, Changsha 410083, China; zenglicsu@csu.edu.cn; 3 Center for Advanced Jet Engineering Technologies (CaJET), School of Mechanical Engineering, Shandong University, 17923 Jingshi Road, Jinan 250061, China; chudongkai@sdu.edu.cn

**Keywords:** MMF interferometer, femtosecond laser, FBG, dual-parameter sensing

## Abstract

A hybrid sensing configuration for simultaneous measurement of strain and temperature based on fiber Bragg grating (FBG) written in an offset multimode fiber (MMF) interferometer using femtosecond laser pulse is proposed and demonstrated. A Mach–Zehnder interferometer is formed by splicing a section of MMF between two single-mode fibers (SMFs) and a high interference fringe of up to 15 dB is achieved. The sensing experimental results show a strain sensitivity of −1.17 pm/με and 0.6498 pm/με for the dip of MZI and Bragg peak, while a temperature sensitivity of 42.84 pm/°C and 19.96 pm/°C is measured. Furthermore, the matrix analysis has found that the strain and temperature resolution of the sensor are as high as ±12.36 με and ±0.35 °C, respectively. In addition, the sensor has merits of simple fabrication, good spectral quality, and high resolution, which shows attractive potential applications in dual-parameter sensing.

## 1. Introduction

Fiber optics sensors have been widely used to measure various physical parameters, such as temperature [1], strain [2], refractive index [3], etc., due to their advantages of anti-electromagnetic interference, high resolution, lightweight, and compact size [3,4,5]. In recent years, various configurations have been proposed for simultaneous measurement of temperature and strain, including various Mach–Zehnder interferometers (MZIs) [6,7,8] and Fabry–Pérot interferometers (FPIs) [9,10,11], fiber Bragg gratings (FBGs) [12], and long-period fiber gratings (LPFGs) [13]. Among them, MZI- or FPI-based devices are usually realized by splicing different special fibers [14,15], micro-cavity [7] fabricated by lasers, and fiber tapers [16,17], which have shown outstanding advantages of higher wavelength sensitivity, simple fabrication, and rich structures. However, the interferometers have too many resonant dips in one wavelength window and similar sensitivity for temperature and strain, making the simultaneous measurement difficult. Moreover, some FBG-based configurations are achieved, including FBGs with different wavelengths [18] and grating types [19], FBGs written in different optical fiber types [20], a tilted FBG [21], and a superstructure FBG [22]. However, the above structures have relatively low measurement accuracy. To solve the above issues of dual parameter sensing, some hybrid structures are proposed, such as Fabry–Perot interferometer cascaded with an FBG [23], FBG-tapered FBG-no core fiber [24], FBG-S fiber taper-FBG [25], cascading MZI with an FBG [26,27] and spheroidal-cavity-overlapped FBG [28]. Those configurations provide an effective approach to distinguish the cross-sensitivity between temperature and strain. However, the resolution of the sensors needs to be further improved.

In this paper, we have proposed and investigated the sensing performances of a novel hybrid scheme for simultaneous measurement of strain and temperature, which consists of an FBG embedded in one MZI based on offset single mode-multimode-single mode (SMS) fiber structure. The theoretical matrix analysis has proved that the measurement accuracy can be improved obviously by taking the FBG wavelength as a sensing peak. The strain and temperature resolution are as high as ±12.36 με and ±0.35 °C, respectively, which is a resolution higher than many fiber sensors based on cascade structure and dual FBGs. In addition, the sensor has the advantages of simple fabrication and high fringe visibility. Those characteristics have displayed the great potential of the sensor in the field of simultaneous measurement of dual parameter sensing.

## 2. Device Fabrication and Sensing Principle

**Figure 1:**
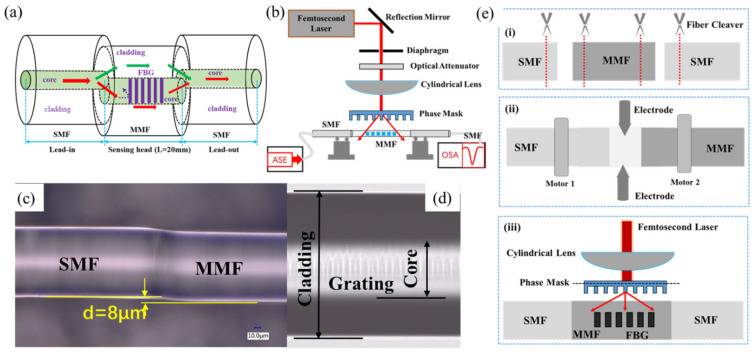
(**a**) The schematic diagram of the hybrid structure. (**b**) The schematic diagram of grating fabrication system with femtosecond laser phase mask method. (**c**) The optical image of the fusion region between SMF and MMF. (**d**) The optical image of the grating modulation region. (**e**) The schematic diagram of the fabrication process of the sensor.

**Figure 2:**
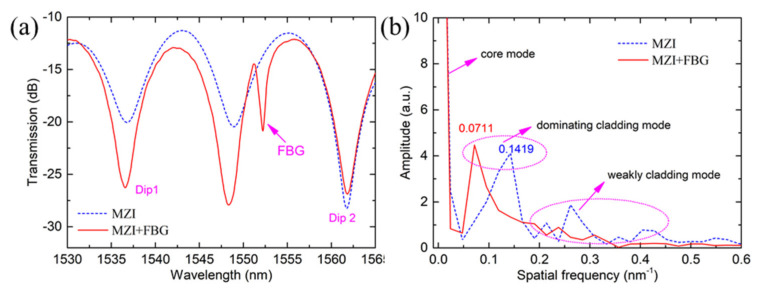
(**a**) The transmission spectrum of the proposed sensor (**b**) The spatial frequency spectrum of the single MZI without FBG and hybrid structure.

Figure 1a shows the schematic diagram of the hybrid structure. The sensor consists of an MZI and an FBG. The MZI is formed by offset splicing a section of MMF (MMF50/125, Nufern) between two SMFs (SMF-28e, Corning) as displayed in our previous work [8]. The core and cladding diameters of the MMF are 50 μm and 125 μm, respectively. The MMF length is set as about 20 mm and the MZI is fabricated using only a fiber cleaver (CT 30, Fujikura) and a fusion splicer (FSM 80 s, Fujikura). Also, the core offset welding is realized by using the “Attenuation Mode (AM)” on the fusion splicer menu. As shown in Figure 1a, the light in the lead-in SMF is divided into two parts, one path of light is transmitted in the core of MMF, the other path is transmitted in the cladding of MMF. At the right fusion offset joint, the light transmitted in the cladding of MMF will be recoupled into the core of SMF. The two paths of light will produce a phase difference in the core of the lead-out fiber due to the difference in the effective refractive index between the core and cladding of MMF and form an interference pattern in the transmission spectrum. FBG induced by femtosecond laser will reflect part of the light transmitted in the core of MMF into the collapsed area at the fusion joint and the transmitted light through the grating will couple with the light transmitted in the cladding of MMF. In addition, the induced FBG changes the refractive indices of the core of MMF, which results in the phase change of MZI, showing a change in transmission intensity of dips. The MZI based on SMS structure is a classical configuration and simple to fabricate. Here, we have used the lateral offset fusion method to increase the excitation of the cladding mode in the MMF, and an offset of about 8 μm between the core of SMF and MMF is achieved, as shown in Figure 1c. The FBG is written by using femtosecond laser phase mask technology, as shown in Figure 1b. The femtosecond laser pulses are generated by an 800 nm sapphire amplifier (Spectra-physics) with a duration of 120 fs and a repetition rate of 1 kHz. The light beam is focused through a cylindrical lens with a focal length of 50 mm into the phase mask and the light beam diameter and energy flux are controlled by a diaphragm an optical attenuator, respectively. The phase mask (Ibsen Photonics) is optimized for an 800 nm femtosecond laser and designed with a period of 2142 nm and zero-order diffraction of below 4%, which can produce 2nd-order Bragg grating at 1550 nm band. The FBG is written in the core of MMF with single pulse energy of 0.6 mJ and an exposure time of about 30 s. The fabrication process of the sensor is described as shown in Figure 1e. The steps are as follows: (i) use fiber cutting plier to remove the fiber coating layer of the optical fibers and clean their fiber surfaces with alcohol, and then use a fiber cleaver to cut a section of MMF with a length of about 20 mm as well as obtain the fiber ends of SMF and MMF; (ii) select the welding mode and use a fusion splicer to discharge and splice SMF and MMF, forming an MZI with SMS structure; (iii) fix the MZI device on the fiber clamp and adjust the laser parameters of phase mask fabrication, and finally make an FBG in the MMF core. Figure 2a shows the transmission spectrum of the single MZI without FBG and the hybrid structure. It can be found that the proposed hybrid sensor has shown deeper dips than the single MZI without FBG and high fringe visibility of up to 15 dB is achieved. This phenomenon could be due to the refractive index change in the core of MMF induced by the introduction of FBG. In addition, broadband amplified spontaneous emission (ASE) source with a wavelength range of 1528–1602 nm and an optical spectrum analyzer (OSA, Agilent 86142B) with a wavelength range 600–1700 nm) and a wavelength resolution of 0.01 nm are connected to record the transmission spectrum as displayed in the Figure 1b.

To analyze the power distribution of the interference modes, the transmission spectrum is transformed into the spatial frequency spectrum by fast Fourier transformed (FFT), as shown in Figure 2b. It can be observed that only one dominant cladding mode for the single MZI without FBG and hybrid structure located at 0.1419 nm^−1^ and 0.0711 nm^−1^ are excited, respectively. The introduction of the FBG leads to higher-order cladding modes for the MZI, which is attributed to refractive index modulation induced by femtosecond laser pulses.

According to light propagation theory, the phase delay ($\Phi_{m}$) for the multimode interference between the core mode and cladding modes can be simple written as [29]:

(1) $$\Phi^{m}=\frac{2\pi \left(n_{eff}^{core}-n_{eff}^{clad,m}\right)L}{\lambda_{m}}=\frac{2\pi \Delta n_{eff}^{m}L}{\lambda_{m}}$$

where $n_{eff}^{core}$ and $n_{eff}^{clad,m}$ are the effective refractive index of core mode and cladding modes, respectively; L and $\lambda_{m}$ are the length of MZI and the resonant wavelength, respectively; $\Delta n_{eff}^{m}$ is the difference of effective refractive index of core and cladding modes. The resonant wavelength can be expressed as [29]:

(2) $$\lambda_{m}=\frac{2\Delta n_{eff}^{m}L}{2m+1}$$

According to the couple-mode theory, the resonant wavelength of FBG can be expressed as:

(3) $$k\lambda_{FBG}=2n_{eff}^{core}\Lambda$$

where Λ is grating period, k is the order of Bragg grating, which is a constant.

When temperature is applied to the sensor, the resonant wavelengths of MZI and FBG could shift due to the change of effective refractive index of core and cladding caused by the thermo-optic effect. Similarly, the elasto-optic effect induced by the applied strain could produce a change in that effective refractive index of core and cladding modes. Therefore, the resonant wavelengths of MZI and FBG will produce a wavelength shift as the temperature and strain increase. The sensor can be used to measure the temperature and strain change by tracking the changes in wavelengths.

## 3. Experiment Results and Discussion

**Figure 3:**
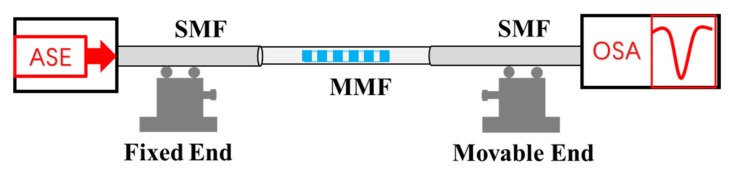
The schematic diagram of the experimental setup of the strain measurement.

**Figure 4:**
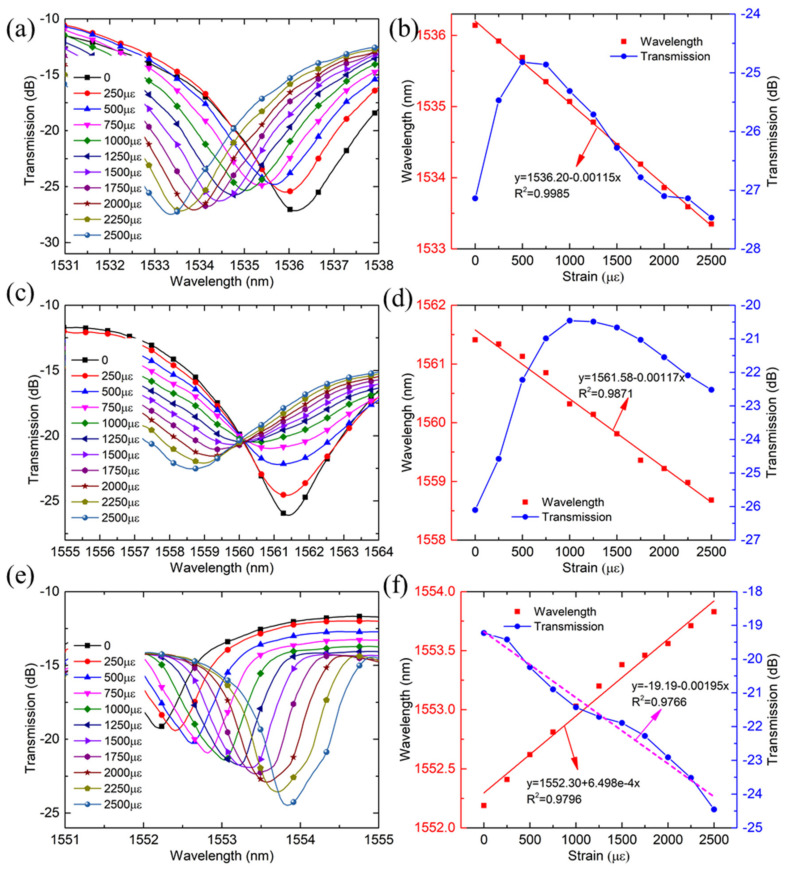
The transmission spectra change as the strain increases for different resonant wavelengths: (**a**) dip1 (**c**) dip2 (**e**) FBG peak, and the wavelength shift and transmission change as a function of strain change for three resonant wavelengths: (**b**) dip1; (**d**) dip 2; (**f**) FBG peak.

During the strain test, the sensor is fixed on two micro-displacement platforms (GCM-127201AM, DaHeng Optics) as shown in Figure 3. The original length of the two platforms is set as 200 mm, and each time the movable displacement platform is stretched forward with a step of 50 μm and the corresponding strain is 250 με. The measurement strain range is set as 0~2500 με. To compare the strain and temperature characteristics of FBG peak and the wavelengths of MZI, two dips of MZI at 1536 nm and 1561 nm and FBG wavelength at 1552 nm are selected, as displayed in Figure 4. Figure 4 shows the transmission spectrum change for three wavelengths as the strain increases. It is found that the dips of MZI have shown a blue-shift, but the FBG peak has a red-shift as the strain increases. Dip 1 and dip 2 have a wavelength variation of 2.79 nm and 2.73 nm in the range of 0~2500 με, respectively, as shown in Figure 4b,d. A strain sensitivity of −1.15 pm/με and −1.17 pm/με for the dip 1 and dip 2 is achieved by linear fitting, respectively. The fitting results have shown a perfect linear relationship between the strain and wavelength shift, and a high goodness of over 0.9985 are obtained. Meanwhile, the FBG peak has a red-shift with a wavelength change of 1.64 nm as the strain increases. The strain sensitivity of 0.6498 pm/με with a goodness of fit of 0.9796 is achieved by linear fitting, as shown in Figure 4f. In addition, the transmission loss of the two dips of MZI shows a tendency to increase before decreasing as displayed in Figure 4b,d. However, the FBG peak has shown a trend of linear reduction as the strain increases, and a transmission loss sensitivity of −0.00195 dB/με is achieved, as displayed in Figure 4f. The transmission loss change of MZI is closely related to the splitting ratio of light, difference of the refractive index change, and the length of MZI [30]. When a strain is applied, the strain-induced changes in the difference of the effective indices are dominant, resulting in the transmission loss change of dip1 and dip2 is significant and those changes are nonlinearity.

**Figure 5:**
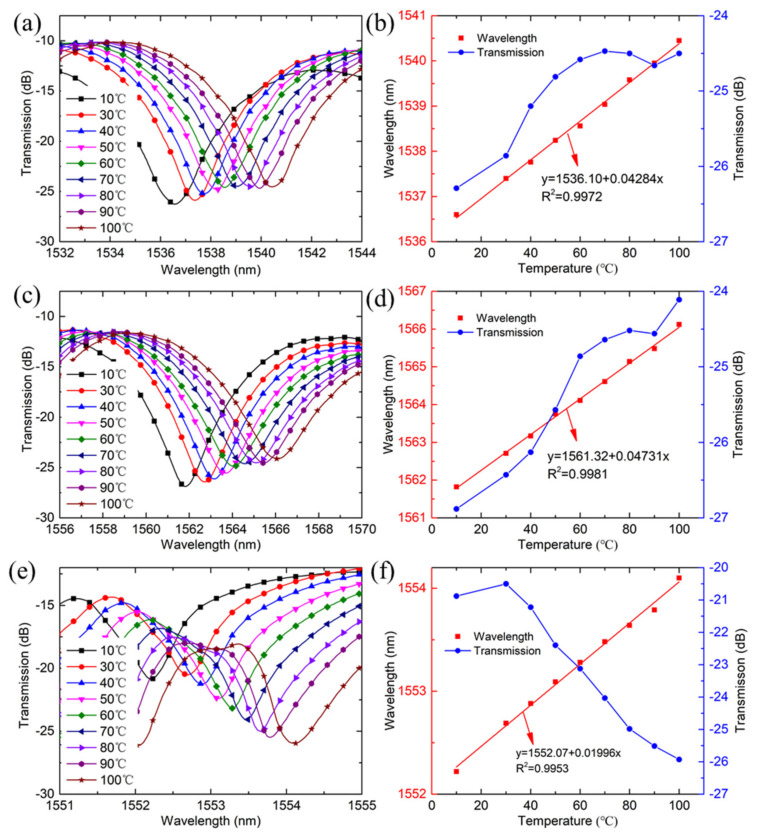
The transmission spectra change as temperature increases for different resonant wavelengths: (**a**) dip 1; (**c**) dip 2; (**e**) FBG peak, and the wavelength shift and transmission change as a function of temperature for three resonant wavelengths: (**b**) dip1; (**d**) dip 2; (**f**) FBG peak.

During the temperature test, the sensor is fixed on an electric heating plate with a resolution of 1 °C. As the temperature increases from room temperature to 100 °C, the three wavelengths present red-shifts, and a wavelength shift of 3.85 nm, 4.3 nm, and 1.88 nm is achieved for the dip1, dip2, and FBG peak, respectively, as shown in Figure 5a,c,e. The wavelengths all display linear responses to the temperatures and a temperature sensitivity of 42.84 pm/°C, 47.31 pm/°C and 19.96 pm/°C for the dip1, dip2 and FBG peak is achieved in the range of 10–100 °C, as shown in Figure 5b,d,f. Moreover, the transmission loss of the dips of MZI and FBG peak have shown an opposite trend. The transmission of dip 1 and dip 2 increases at an average rate of 0.0199 dB/°C and 0.0308 dB/°C, respectively, while the FBG decreases at a rate of 0.0811 dB/°C as the temperature increases.

**Figure 6:**
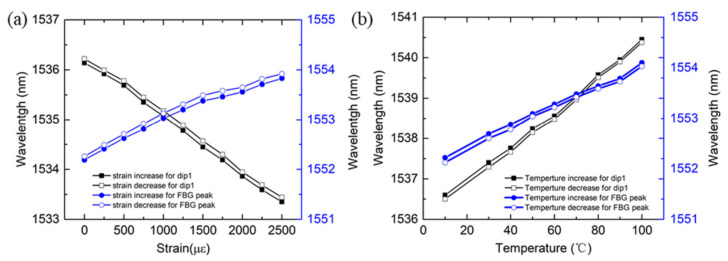
The wavelength changes of dip1 and FBG peak as the (**a**) strain and (**b**) temperature increases and decreases.

In addition, the wavelength hysteresis of the sensor induced by strain and temperature are investigated as shown in Figure 6. The strain measurement is conducted as the strain increases from 0 to 2500 με and decreases from 2500 με to 0, and it can be found that the max wavelength hysteresis error of the dip1 and FBG peak is about 0.12 nm and 0.11 nm, respectively, as shown in Figure 6a. A wavelength of hysteresis of 0.11 nm and 0.1 nm for the dip1 and FBG peak, respectively, is observed as shown in Figure 6b, as the temperature increases from 10 °C to 100 °C and decreases from 100 °C to 10 °C. From the investigation, it can be found that the dips of MZI and FBG peaks have similar wavelength hysteresis.

When the strain and temperature are applied to the sensor simultaneously, the wavelength shifts of dip1 and FBG peak can be simply expressed by the matrix demodulation method as follows [31]:

(4) $$\left[\begin{array}{l} \Delta \lambda_{1}\\ \Delta \lambda_{2} \end{array}\right]=\left[\begin{array}{cc} K_{1T} & K_{1\varepsilon }\\ K_{2T} & K_{2\varepsilon } \end{array}\right]\left[\begin{array}{l} \Delta T\\ \Delta \varepsilon \end{array}\right]$$

where $\Delta \lambda_{1}$ and $\Delta \lambda_{2}$ is the wavelength shift of dip1 and FBG peak, respectively; $K_{1T}$, $K_{2T}$ $K_{1\varepsilon }$, $K_{2\varepsilon }$ are the temperature and strain sensitivities of the dips of the MZI and FBG peaks; ΔT and Δε is the temperature and strain variations, respectively. Thus, the temperature and strain measurement matrix can be written as follows [31]:

(5) $$\left[\begin{array}{l} \Delta T\\ \Delta \varepsilon \end{array}\right]=\frac{1}{D}\left[\begin{array}{cc} K_{2\varepsilon } & -K_{1\varepsilon }\\ -K_{2T} & K_{1T} \end{array}\right]\left[\begin{array}{l} \Delta \lambda_{1}\\ \Delta \lambda_{2} \end{array}\right]$$

where $D=\left|K_{1T}K_{2\varepsilon }-K_{1\varepsilon }K_{2T}\right|$ is the absolute value of the coefficient matrix.

Assume the wavelength resolution of two dips are $\delta (\Delta \lambda_{1})$ and $\delta (\Delta \lambda_{2})$, respectively. Theoretically, the temperature and strain resolution (δ(ΔT) and δ(Δε)) can be written as follows [31]:

(6) $$\left[\begin{array}{l} \delta (\Delta T)\\ \delta (\Delta \varepsilon ) \end{array}\right]=\pm \frac{1}{D}\left[\begin{array}{cc} \left|K_{2\varepsilon }\right| & \left|K_{1\varepsilon }\right|\\ \left|K_{2T}\right| & \left|K_{1T}\right| \end{array}\right]\left[\begin{array}{l} \delta (\Delta \lambda_{1})\\ \delta (\Delta \lambda_{2}) \end{array}\right]$$

According to Equation (5), the simultaneous measurement of temperature and strain can be achieved by using the dip1 and FBG peak, and changes of dual parameters can be expressed as follows:

(7) $$\left[\begin{array}{l} \Delta T\\ \Delta \varepsilon \end{array}\right]=\frac{1}{50.79}\left[\begin{array}{cc} 0.6498 & 1.15\\ -19.96 & 42.84 \end{array}\right]\left[\begin{array}{l} \Delta \lambda_{1}\\ \Delta \lambda_{2} \end{array}\right]$$

As the OSA has a resolution of 10 pm, according to Equation (6), the temperature and strain resolution can be written as follows:

(8) $$\left[\begin{array}{l} \delta (\Delta T)\\ \delta (\Delta \varepsilon ) \end{array}\right]=\pm \frac{1}{50.79}\left[\begin{array}{cc} 0.6498 & 1.15\\ 19.96 & 42.84 \end{array}\right]\left[\begin{array}{l} 10\\ 10 \end{array}\right]=\pm \left[\begin{array}{l} 0.35\\ 12.36 \end{array}\right]$$

In addition, through the above analysis, if the dip1 and dip2 are selected as sensing peaks, the corresponding temperature and strain measurement matrix can be given as

(9) $$\left[\begin{array}{l} \Delta T\\ \Delta \varepsilon \end{array}\right]=\frac{1}{4.28}\left[\begin{array}{cc} -1.17 & 1.15\\ -47.31 & 42.84 \end{array}\right]\left[\begin{array}{l} \Delta \lambda_{dip1}\\ \Delta \lambda_{dip2} \end{array}\right]$$

Thus, the corresponding temperature and strain resolution can be written as follows:

(10) $$\left[\begin{array}{l} \Delta T\\ \Delta \varepsilon \end{array}\right]=\pm \frac{1}{4.28}\left[\begin{array}{cc} 1.17 & 1.15\\ 47.31 & 42.84 \end{array}\right]\left[\begin{array}{l} 10\\ 10 \end{array}\right]=\pm \left[\begin{array}{l} 5.42\\ 210.45 \end{array}\right]$$

According to the Equations (8) and (10), it can be seen that the temperature and strain resolution are calculated by using the dip1 and FBG peak are ±0.35 °C and ±12.36 με, respectively, which is a resolution significantly higher than that by dip 1 and dip 2. The introduction of the FBG in the SMS sensing structure enhances the measurement resolution obviously due to the increase of D in Equation (6). The determination error of peak wavelength can be lower than the resolution of OSA and depends on the determination algorithm. Therefore, the resolution of this sensor can be enhanced further through some determination algorithms [32,33].

In addition, the sensing performances of different structures based on FBG and MZI are displayed in Table 1. The sensor we reported has shown higher temperature sensitivity and strain sensitivity than that of the dual-parameter sensors based on dual FBGs, cascade structures, and FBG written in some special fibers such as POFs and Bragg fiber. The dual gratings and cascade structures are the most common configurations and are simple to fabricate. However, the dual FBGs have similar temperature and stain sensitivity, which is difficult to distinguish between temperature and strain simultaneously. And, the cascade structures increase the length of the device, which will produce larger measurement errors caused by bending or refractive index. The FBGs written in DMF and Bragg fibers increase the complexity and cost of fabrication, and that written in POFs have larger insertion loss and relatively poor spectrum quality. Although the FBGs modified with reduced graphene oxide films have higher strain sensitivity, about 5 times than that of bare FBG, the coating technology is complex and the FBG device needs corrosion treatment, which reduces the mechanical strength. From the investigation, the hybrid sensor we proposed has shown significant advantages of simple fabrication, high resolution, and relatively small size. Those demonstrate its great potential in the application of dual-parameter simultaneous sensing.

## 4. Conclusions

In conclusion, a high sensitivity sensor based on a hybrid structure for simultaneous measurement of strain and temperature is proposed and experimentally demonstrated. The sensor consists of an MZI based on SMS structure and an FBG fabricated by the femtosecond laser phase mask method. The sensor has shown a strain sensitivity of −1.15 pm/με and 0.6498 pm/με, as well as a temperature sensitivity of 42.84 pm/με and 19.96 pm/με for dip1 of MZI and FBG peak, respectively. Moreover, the temperature and strain resolution are up to ±0.35 °C and ±12.36 με through the demodulation matrix analysis, which is a resolution significantly higher than some reported configurations, such as cascade structure based on MZI and FBG, fiber gratings, and various MZIs. In addition, the proposed sensor has advantages of easy fabrication and relatively high resolution, which has shown great potential for simultaneous measurement of strain and temperature in the fields of dual-parameter sensing.

## Figures and Tables

**Table 1:** Performance comparison of different fiber sensing structures.

Structure Types	Temperature Sensitivity	Strain Sensitivity	Measurement Resolution	References
Cascade structure with MZI and FBG	9 pm/°C (FBG); 52 pm/°C (MZI)	0.48 pm/με (FBG); −0.45 pm/με (MZI)	/	[26]
Cascade structure with FPI and FBG	0.162 pm/°C (FPI) 7.82 pm/°C (FBG)	2.1 pm/με (FPI) 1.01 pm/με (FBG)	/	[23]
Dual FBGs	11.4 pm/°C (FBG1) 15.2 pm/°C (FBG2)	0.22 pm/με (FBG1) 0.24 pm/με (FBG2)	28.3 με 4.1 °C	[18]
FBGs written in DMF	7.9 pm/°C (LP01); 9.3 pm/°C (LP11)	1.21 pm/με (LP01); 1.24 pm/με (LP01)	±0.8 °C ±6.3 με	[34]
Reduced graphene oxide coated etched FBGs	33 pm/°C	5.5 pm/με	1 με 0.3 °C	[2]
Multimode POF-FBG	102.2 pm/°C (MZI) −64.6 pm/°C (FBG)	−3.03 pm/με (MZI) 1.51 pm/με (FBG)	±1.1 °C ±40.3 με	[35]
FBG written in Bragg fiber	9.68 pm/°C (dip A) 11.2 pm/°C (dip D)	1.10 pm/με (dipA) 1.12 pm/με (dipD)	±1.2 °C ±26 με	[36]
Hybrid structure with MZI and FBG	19.96 pm/°C (FBG); 42.84 pm/°C (MZI)	0.65 pm/με (FBG); −1.15 pm/με (MZI)	±0.35 °C ±12.36 με	our work
